# Clinical and genetic analysis of *MOCOS* gene-related hypouricemia

**DOI:** 10.3389/fgene.2025.1636032

**Published:** 2025-10-15

**Authors:** Huifang Peng, Ping Tu, Qiaobo Ma, Jinpeng Tang, Wannv Xiao, Xinyu Hu, Yingyu Zhang, Hongwei Jiang

**Affiliations:** ^1^ Henan Key Laboratory of Rare Diseases, Endocrinology and Metabolism Center, The First Affiliated Hospital, and College of Clinical Medicine of Henan University of Science and Technology, Luoyang, China; ^2^ Endocrinology Department, Nanchang People’s Hospital, Nanchang, China; ^3^ Graduate School of Jiangxi University of Traditional Chinese Medicine, Nanchang, China

**Keywords:** xanthinuria type II, hypouricemia, nephrolithiasis, MOCOS, rare disease

## Abstract

**Background:**

Uric acid is an important metabolic end-product in the human body, and metabolic abnormalities involving uric acid are receiving increasing attention.

**Methods:**

This study involved clinical assessment and genetic testing of a 40-year-old male patient who presented with the main complaint of hypouricemia for 7 years.

**Results:**

In addition to hypouricemia, the patient showed low urinary uric acid levels, low uric acid excretion rate, and nephrolithiasis. His younger brother also showed extremely low serum uric acid levels. Trio-whole-genome sequencing (trio-WGS) showed that the proband and his younger brother had a compound heterozygous molybdenum cofactor sulfurase (MOCOS) genotype with the pathogenic variant c.1272T>A (p.Cys424Ter) and the likely pathogenic variant c.1418C>T (p.Ser473Leu). The final diagnosis was Xanthinuria type II. A review of the literature for cases of Xanthinuria type II revealed reports describing 25 patients from 17 families. All 25 patients showed very low serum uric acid levels, eight showed urinary tract stones, and three reported joint or limb pain. Truncation pathogenic or likely pathogenic variants of the *MOCOS* gene accounted for nearly half of the cases, and p.Arg419Ter and p.Thr349Ile were the two most frequent variants. The p.Cys424Ter variant reported in this study is a new pathogenic site that has not been reported previously.

**Conclusion:**

Sustained low serum uric acid levels may indicate monogenic uric acid metabolism disorders, and these patients should undergo genetic testing. In patients diagnosed with xanthinuria type II caused by *MOCOS* gene variants, the use of purine drugs should be prohibited to avoid serious adverse events. Given the severe defects in the *MOCOS* gene-deficient mouse model, additional research is needed to clarify the clinical profile of Xanthinuria type II and the other roles of MOCOS in metabolic pathways.

## 1 Introduction

In humans, uric acid is primarily synthesized from purines in the liver or intestines through xanthine dehydrogenase (XDH; EC 1.17.1.4 in the Expasy database) and reactive oxygen species (ROS)-dependent pathways, followed by renal or intestinal excretion. Molybdenum cofactor sulfurase (MOCOS; EC 2.8.1.9) sulfurates both XDH and aldehyde oxidase (AOX1, EC 1.2.99.7), which is essential for the activation of these two enzymes (Ichida et al., 2001). Ribose-5-phosphate (Ribose-5-P) and adenosine triphosphate (ATP) are converted into phosphoribosyl pyrophosphate (PRPP) by synthetase, which subsequently generates inosine monophosphate (IMP) and inosine. Inosine is then catalyzed by purine nucleoside phosphorylase (PNP; EC 2.4.2.1) to form hypoxanthine, which further contributes to purine synthesis. Fructose metabolism, protein degradation, alcohol metabolism, and severe tissue hypoxia can all increase purine production, while dietary intake also contributes to purine accumulation in the body (Wen et al., 2024), and hyperuricemia is frequently encountered in clinical practice. The hypouricemia is defined by serum uric acid levels below 2 mg/dL (119 μmol/L). Aside from drug-induced causes, reduced synthesis or increased excretion of uric acid can lead to hypouricemia, with genetic defects accounting for a minority of the cases. Xanthinuria type I (caused by *XDH* gene defects, OMIM#278300) or type II (*MOCOS* gene defects, OMIM#603592) as well as purine nucleoside phosphorylase deficiency (*PNP* gene defects, OMIM#613179) are forms of hypouricemia resulting from impaired synthesis pathways. All of these three conditions are characterized by extreme hypouricemia and low, even undetectable, urinary uric acid levels, with hypouricemia being the key diagnostic feature. Some patients may also show markedly elevated urinary xanthine concentrations (>25 μmol/mol creatinine). More than 50% of patients with hereditary xanthinuria remain asymptomatic (Sebesta et al., 2018). Although renal hypouricemia type I (solute carrier family 22, member 12 [*SLC22A12*] gene defects, OMIM#220150) or type II (solute carrier family 2, member 9 [*SLC2A9*] gene defects, OMIM#612076) have been reported to arise from defective renal tubular uric acid reabsorption, leading to excessive excretion of uric acid (Nakayama et al., 2023), monogenic-related hypouricemia is extremely rare. In this study, we conducted clinical analysis and genetic testing of a Chinese family with extremely low serum uric acid levels. Additionally, we have summarized the clinical characteristics and genetic variants of previously reported cases of *MOCOS* defect-associated Xanthinuria type II to enhance the understanding of the diagnosis and treatment of hypouricemia.

## 2 Materials and methods

Written informed consent was obtained from the patient, and approval was obtained from the Ethics Committee of the First Affiliated Hospital of Henan University of Science and Technology.

### 2.1 Clinical data

The male proband, who sought medical attention due to hypouricemia, underwent systematic medical examinations, including assessment of medical history, physical examination (measurement of height, weight, waist-to-hip ratio, blood pressure, heart rate, etc.), biochemical examinations (liver function, kidney function, thyroid function, myocardial enzyme spectrum, blood lipids, blood glucose, and routine blood and urine examinations), imaging examination (ultrasound examination, chest computed tomography (CT) cranial magnetic resonance imaging, electrocardiogram, etc.), and investigation of family history.

### 2.2 Genetic testing

Peripheral blood samples (2 mL) of the proband, his parents, and his younger brother were collected for trio-whole genome sequencing (trio-WGS) using DNBSEQ-T7 (BGI, China). Suspected variants were validated by Sanger sequencing, and evaluation of variant pathogenicity was conducted in accordance with the variant pathogenicity guidelines established by the American College of Medical Genetics and Genomics/Association for Molecular Pathology (ACMG/AMP).

### 2.3 Bioinformatics analysis of variants

The hydrophobicity in the wild-type and variant were predicted using Expasy ProtScale database (https://web.expasy.org/protscale/) to analyze the hydrophobicity of amino acids and their surrounding amino acids. The Adaptive Poisson Boltzmann Solver (APBS) plugin in ChimeraX (https://www.cgl.ucsf.edu/chimerax/) was used to analyze the changes in the surface electrostatic potential of the proteins with and without the mutation. Analysis of the influence of suspected missense variations on protein tertiary structure was performed using Alphafold3 (https://golgi.sandbox.google.com/) for modeling and the PyMOL Molecular Graphics System Version2.0 for mapping. The stability of the mutated proteins was analyzed using DUET (http://biosig.unimelb.edu.au/duet/stability), MUpro (https://mupro.proteomics.ics.uci.edu/), DynaMut2 (https://biosig.lab.uq.edu.au/dynamut2/submit_prediction_mm), SAAFEC-SEQ (http://compbio.clemson.edu/SAAFEC-SEQ/#started), and I-Mutant2.0 SEQ (https://folding.biofold.org/i-mutant/i-mutant2.0.html). The output results were expressed as ΔΔG (kcal/mol), with negative values indicating a reduction in protein stability and suggesting that the mutation affected protein stability.

### 2.4 Review of published cases

Literature databases (including PubMed, Medline, Embase, and CNKI) were reviewed to summarize all cases of xanthinuria caused by pathogenic or likely pathogenic variants of the *MOCOS* gene and analyze the clinical characteristics and genotypes.

## 3 Results

### 3.1 Clinical manifestations

**FIGURE 1:**
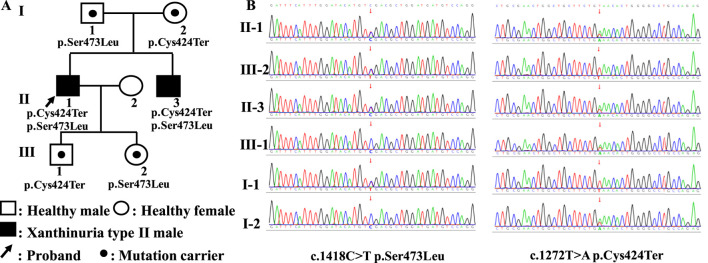
Family tree and the results of Sanger sequencing. **(A)** The family tree of the proband in this study. **(B)** The results of Sanger sequencing of the *MOCOS* c.1272T>A (p.Cys424Ter) and c.1418C>T (p.Ser473Leu) variants.

The proband was a 40-year-old man who presented with the main complaint of hypouricemia for 7 years. A physical examination conducted 7 years previously showed serum uric acid levels of 4.00 μmol/L (reference value, 208.00–428.00 μmol/L). Subsequent tests showed serum uric acid levels between 0 and 11.00 μmol/L. However, since the patient experienced no other related discomfort, the low serum uric acid levels were not seriously. He had been diagnosed with hepatitis B virus (HBV) infection at 21 years of age and was treated with lamivudine. The physical examination yielded the following findings: height, 162 cm; weight, 71 kg; body mass index (BMI), 27 kg/m^2^; waist circumference, 94 cm; hip circumference, 98 cm; waist-to-hip ratio, 0.96; blood pressure, 66/116 mmHg; heart rate, 66 beats/min; and no abnormalities on internal palpation. The laboratory tests yielded the following findings: urea nitrogen, 5.40 mmol/L (2.90–8.20 mmol/L); creatinine, 93.00 (59.00–104.00 μmol/L); serum uric acid, 12.00 μmol/L (208.00–428.00 μmol/L); urine uric acid, 10.00 μmol/L (210.00–420.00 μmol/L); urine creatinine, 10,388.00 μmol/L (7,000.00–18000.00 μmol/L); calculated 24-h urinary uric acid excretion rate, 0.74%. The patient showed no significant abnormalities in eight liver function parameters, thyroid function parameters, myocardial enzyme spectrum, four blood lipid parameters, blood glucose levels, blood routine examination, and urine routine examination. The ultrasound examination indicated the presence of 2 mm × 2 mm stones in the left kidney, with no abnormalities in ultrasound assessments of the liver, gallbladder, pancreas, spleen, thyroid, heart, and carotid artery. Chest CT showed small nodules in the lungs. Head magnetic resonance imaging (MRI) and electrocardiogram showed no abnormalities. The nephrolithiasis spontaneously passed through in the next 2 days and was not captured, precluding analysis of the composition of the stone. Assessment of the patient’s family history showed that the proband’s younger brother had hypouricemia and HBV infection. Unfortunately, we did not obtain the test results for uric acid, xanthine, and hypoxanthine, which posed a limitation for the diagnosis. The patient’s father was in good health, while his mother had diabetes and atrial fibrillation; the parents’ marriage was non-consanguineous. The proband had one son and one daughter in good health (Figure 1A).

### 3.2 Trio-WGS results

Compound heterozygous variants of the *MOCOS* gene (NM_017947.4) were detected in the proband and his younger brother, including c.1272T>A (p.Cys424Ter) located at chr18:36213419 (GRCh38) and c.1418C>T (p.Ser473Leu) located at chr18:36215598, which were inherited from their mother and father, respectively. The ACMG standards and guidelines classify the c.1272T>A (p.Cys424Ter) variant as pathogenic, and the detailed evidence is as follows: PVS1, a nonsense variant in the *MOCOS* gene where loss of function (LOF) is the mechanism underlying Xanthinuria type II; PM2_Supporting, the c.1272T>A variant is not included in the gnomAD database; and PP1_Supporting, segregation in two affected individuals in this family. The c.1418C>T (p.Ser473Leu) variant was considered to be likely pathogenic, and the detailed evidence is presented in the following sentences. PM2_Supporting: the c.1418C>T variant showed an extremely low frequency in the gnomAD database (total allele frequency, 0.00001315). PM3: Xanthinuria type II is an autosomal recessive inheritance disease caused by MOCOS variants, and in this case, we identified the c.1272T>A (p.Cys424Ter) variant as the LP, which was located in *trans* and confirmed using trio-WGS and Sanger sequencing in the patient’s parents and his children. PP3: prediction of variant harmfulness *in silico* was performed using SIFT, Polyphen2, AlphaMissense, CADD, and REVEL (score, 0.912), which suggested that the c.1418C>T variant has a deleterious effect on MOCOS. PP1_Supporting: segregation was observed in the two affected individuals in this family. PP4: the proband’s phenotype and family history were highly specific for a disease with a single genetic etiology. The Sanger sequencing results of the *MOCOS* gene are shown in Figure 1B.

### 3.3 *In silico* analysis of the MOCOS p.Ser473Leu variant

**FIGURE 2:**
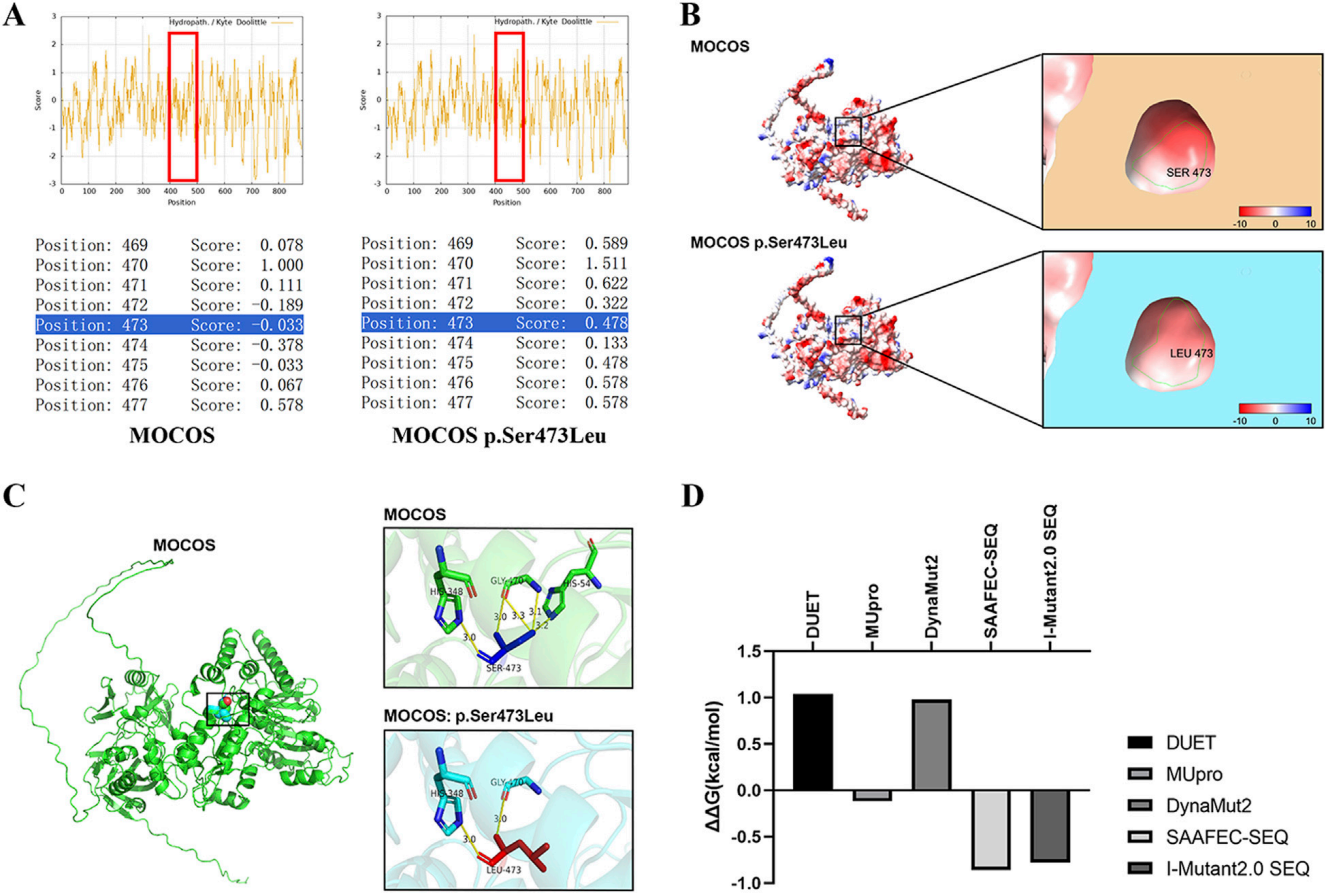
*In silico* analysis of the *MOCOS* p. Ser473Leu variant. **(A)** The Expasy ProtScale database was used to predict the influence of the p. Ser473Leu variant on hydrophilicity, with scores>0 indicating hydrophobicity and scores<0 indicating hydrophilicity. **(B)** Prediction of surface electrostatic potential in the wild-type and the p. Ser473Leu variant using ChimeraX. Blue represents the positive-charge distribution area, red represents the negative-charge distribution area, and white represents the uncharged area. **(C)** Alphafold3 and PyMOL were used to predict the influence of the p. Ser473Leu variant on the tertiary structure of MOCOS. **(D)** Analysis of the effects of the p. Ser473Leu variant on the stability of the MOCOS protein using DUET, MUpro, DynaMut2, SAAFEC-SEQ, and I-Mutant2.0 SEQ. Negative values indicate decreased protein stability.

The Expasy ProtScale database analysis indicated that the Ser473 of MOCOS may interact with water molecules or other hydrophilic motifs inside the protein to help maintain the local conformation of the MOCOS protein. However, in the variant, the hydrophobic amino acid Leu473 disrupt the original hydrophilic interaction, causing the site to aggregate with other hydrophobic regions (Figure 2A). The surface electrostatic potential of the amino acids in the wild-type and the variant showed a negative charge (red area) with no significant change, indicating that the mutation may not affect the surface electrostatic potential of the MOCOS protein (Figure 2B). Modeling of the tertiary structure showed that after the mutation, the distance of hydrogen bonds formed between the main chain Leu473 and His348, Gly470 did not change, while the hydrogen bonds formed between the side chain Leu473 and Gly470, His54 broke, indicating that the p. Ser473Leu variant affects the main chain structure of MOCOS and has a major influence on the tertiary structure of the MOCOS protein (Figure 2C). MUpro, SAAFEC-SEQ and I-Mutant2.0 SEQ predicted a decrease in MOCOS protein stability as a result of the p. Ser473Leu variant (Figure 2D). In summary, *in silico* predictions indicated that the Ser473Leu variant affects the secondary and tertiary structures, hydrophilicity, and stability of the MOCOS protein.

### 3.4 Diagnosis

We diagnosed Xanthinuria type II mainly on the basis of following findings: (1) the clinical manifestations of the patient included extreme hypouricemia, low urinary uric acid level, low uric acid excretion rate, and nephrolithiasis; (2) the patient showed a compound heterozygous *MOCOS* genotype.

Notably, although the serum and urine levels of xanthine and hypoxanthine as well as the activity of XDH, AOX1, and MOCOS are important diagnostic criteria for Xanthinuria type II, these tests cannot be performed in most hospital laboratories.

### 3.5 MOCOS genotypes and related clinical symptoms

A total of eight articles that included 25 patients from 17 families were reported. The 25 patients included 12 males and 13 females, and their ages at diagnosis ranged from 2 years to 8 months to 70 years. All 25 patients had extremely low serum uric acid levels, and eight had kidney or urinary tract stones, which occurred in childhood or middle age. One patient showed polycystic kidney disease and chronic renal failure, while three patients reported joint and limb pain. One patient showed joint pain, hand and foot stiffness, skin necrotizing vasculitis, and an azathioprine (AZA)-induced decrease in the whole blood cell count, which recovered after replacing the medication. The patients also showed other diseases, including type 1 diabetes mellitus (T1DM) or type 2 diabetes mellitus (T2DM), aortic dissecting aneurysm, autoimmune syndrome, dyslipidemia, osteoporosis, cholecystitis, and cancer. Four patients had died at the time of the publication of their respective reports: The patient in Case 1 died due to a ruptured aortic dissection at 70 years of age. The patient in Case 8 died at 61 years of age due to tumors and various metabolic diseases. The patient in Case 9 died at 72 years of age. The patient in Case 10 died at 85 years of age due to cancer and metabolic diseases.

Of the 25 cases, 24 showed compound heterozygous or homozygous pathogenic or likely pathogenic variants in the *MOCOS* gene, while the remaining patient showed only one heterozygous pathogenic variant of the *MOCOS* gene. A total of 15 pathogenic or likely pathogenic variants were identified in the *MOCOS* gene, including four nonsense variants (p.Glu37Ter, p. Arg419Ter, p. Cys424Ter, and p. Arg823Ter), two frameshift variants (p.Gln347fsTer379 and p. Leu363ProfsTer16), eight missense variants (p.Arg98Cys, p. Thr121Met, p. Ala156Pro, p. Thr349Ile, p. Ser473Leu, p. Pro591Ser, p. Arg614Gln, and p. Arg776Cys), and one splice variant (c.942-1G>A). Among them, the frequencies of p. Arg419Ter and p. Thr349Ile were the highest (Table 1).

**TABLE 1:** Summary of MOCOS genotypes and clinical phenotypes from reported cases of Xanthinuria type II.

Case	Year of publication	Sex	Age	Serum uric acid level	Serum creatinine level	Urine uric acid level	Serum hypoxanthine level	Serum xanthine level	Renal calculus	MOCOS variants	Ref.
1^a^	2001	F	66	11.90	406.64	0	19.10	138.05	no	p.Arg419Ter, hom	Ichida et al., 2001
2	2001	M	45	5.95	NR	0	3.67	11.18	no	p.Arg419Ter, hom	Ichida et al., 2001
3	2003	F	43	5.30	NR	NR	8.20	12.20	no	p.Ala156Pro, hom	Yamamoto et al., 2003
4^b^	2007	F	2.67	5.95	NR	NR	NR	NR	yes	p.Arg776Cys, hom	Peretz et al., 2007
5^c^	2007	F	56	23.80	NR	NR	0	0	no	p.Arg776Cys, het p.Gln347fsTer379, het	Peretz et al., 2007
6	2015	M	42	2.10	NR	NR	NR	NR	no	p.Arg419Ter, het	Zhou et al., 2015
7^d^	2018	F	38	0	NR	NR	NR	NR	no	p.Thr121Met, hom	Stiburkova et al., 2018
8^e^	2021	M	41	NR	NR	0	NR	NR	yes	p.Thr349Ile, hom	Peretz et al., 2021
9^f^	2021	F	69	0	NR	0	NR	NR	no	p.Thr349Ile, hom	Peretz et al., 2021
10^g^	2021	M	70	5.95	NR	59.50	NR	NR	no	p.Thr349Ile, hom	Peretz et al., 2021
11	2021	F	46	35.70	NR	11.90	NR	NR	no	p.Leu363ProfsTer16, het; p.Pro591Ser, het	Peretz et al., 2021
12	2022	F	4	5.95	NR	NR	NR	NR	yes	p.Leu363ProfsTer16, hom	Mandal et al., 2022
13	2022	M	4	5.95	NR	NR	NR	NR	yes	p.Arg419Ter, het; p.Arg98Cys, het	Mandal et al., 2022
14	2022	M	12	5.95	NR	NR	NR	NR	yes	c.942-1G>A, het; p.Arg614Gln, het	Mandal et al., 2022
15	2023	F	3	11.90	27.40	NR	NR	NR	yes	p.Arg823Ter,hom	Aydin et al., 2023a; Aydin et al., 2023b
16	2024	F	46	0.67	55.00	0	17.65	8.00	no	p.Glu37Ter, hom	Li et al. (2024)
17	2025	M	40	4.00	93.00	NR	NR	NR	yes	p.Cys424Ter, het; p.Ser473Leu, het	This study
Reference	—	—	—	208.00–428.00 μmol/L	59.00–104.00 μmol/L	210.00–420.00 μmol/L	1.10–3.00 μmol/L	0.70–1.20 μmol/L	—	—	—

^a^ The patient had polycystic kidney disease, chronic renal failure, concomitant aortic dissection aneurysm and died at 70 years of age due to rupture of the aneurysm.

^b^ The proband also reported lower-limb pain; four members of this family were affected by the disease, including two older sisters and one older brother. Among them, one older sister experienced recurrent lower-limb pain from 13 years of age.

^c^ Two members of this family were diagnosed with the disease, and the older brother of the proband developed renal calculus at 35 years of age; the patient’s parents had a consanguineous marriage.

^d^ The patient experienced joint pain, stiff hands and feet, and necrotizing vasculitis of the skin; adverse reactions to azathioprine drugs could have caused the reduction in the whole blood cell count.

^e^ The patient died at 61 years of age. Other diseases in this patient included autoimmune overlap syndrome, non-small cell lung cancer, type 1 diabetes mellitus, abnormal blood lipid levels, cholecystitis, and osteoporosis. This family had four patients, including two older brothers and one younger sister of the proband.

^f^ This patient died at 72 years of age. The patient’s other illnesses included obesity and type 2 diabetes mellitus (T2DM).

^g^ The patient died at 85 years of age, and this patient’s other illnesses included T2DM, metastatic transitional cell carcinoma of the bladder, colon cancer, transient ischemic attack and end-stage renal disease. NR, Not reported.

Notably, Xanthinuria type II caused by pathogenic or likely pathogenic variants of the *MOCOS* gene is a very rare disease, and most of the previous reports were case studies, with no cohort studies. Only 25 cases have been reported to date, precluding a systematic review. Due to the limitations of non-systematic reviews, more research is needed to provide an overview of the disease.

## 4 Discussion

The human *MOCOS* gene is a 56-kb gene located at 18q12.2 and contains 15 exons. The MOCOS protein belongs to the class-V pyridoxal-phosphate-dependent aminotransferase family and consists of 888 amino acids. The 50–481 region of the protein is the aminotransferase class-V domain, with the Cys amino acid at position 424 being the activation site; the 706–867 region is the molybdenum cofactor sulfatase C-terminal (MOSC) domain, which is a sulfur-loaded domain that receives sulfur extracted by phosphopyridoxal-dependent NifS-like enzymes and delivers it to the enzyme active centers of XDH and AOX1 for activation. Pathogenic variations of the *MOCOS* gene yield proteins lacking the ability to activate XDH and AOX1, blocking the conversion of xanthine to uric acid. The resultant condition, Xanthinuria type II, is characterized by extremely low serum uric acid levels, elevated xanthine and hypoxanthine levels, and nephrolithiasis. It shows autosomal recessive inheritance, and homozygous variants can be detected in consanguineous families (Peretz et al., 2007).

Patients with Xanthinuria type II usually seek medical attention with extremely low serum uric acid levels, and their urine also shows low uric acid and high xanthine levels. In the reported cases, the serum uric acid levels were 0–35.70 μmol/L, which is very low. Although some patients may live without related symptoms until middle age and be eventually diagnosed due to the accidental discovery of low serum uric acid levels, some patients may experience joint pain, nephrolithiasis, and other symptoms during childhood. Therefore, extremely low serum uric acid levels in individuals of any age should be taken seriously and investigated further. The concentrations of xanthine in urine and hypoxanthine in serum and urine as well as evaluations of XDH and MOCOS activity can facilitate the diagnosis of xanthinuria type I or type II. Genetic testing is also very important for the diagnosis of xanthinuria or extreme hypouricemia.

Nephrolithiasis or urinary tract stones (formed due to xanthine accumulation) have been described in eight cases with *MOCOS* gene defects. The incidence of nephrolithiasis associated with monogenic diseases is approximately 20% (333/1,675), including 26% in children and 8% in adults. Xanthinuria type II accounts for 2.1% of the pediatric cases of monogenic nephrolithiasis (Spasiano et al., 2024). In one study, among 54 children (age, <18 years) with nephrolithiasis who underwent whole-exome sequencing (WES) analysis, six received a clear molecular diagnosis. Among these six patients, three had causative *MOCOS* gene pathogenic or likely pathogenic variants; these included a 4-year-old girl, a 4-year-old boy, and a 12-year-old boy showing clinical manifestations such as bilateral nephrolithiasis and extremely low serum uric acid levels (0.1 mg/dL, 5.95 μmol/L) (Mandal et al., 2022). One report described a 3-year-old girl with Xanthinuria type II who had undergone four surgical treatments for nephrolithiasis, including two rounds each of ureteroscopic lithotripsy and nephrolithotomy (Aydin et al., 2023a; Aydin et al., 2023b). Identifying the composition of the nephrolithiasis can facilitate the diagnosis. Unfortunately, in this study, the nephrolithiasis spontaneously passed through in the next 2 days and was not captured, precluding characterization of the stone composition. Although the patient’s nephrolithiasis not require lithotripsy, he was informed that the risk of recurrent urinary stones was very high, and that he may require surgical lithotripsy (ureteroscopic lithotripsy or nephrolithotomy).

At present, therapeutic management of patients with Xanthinuria type II is mainly based on a low-purine diet, with regular monitoring of urinary tract stones and timely symptomatic treatment. However, effective data on specific therapeutic modalities and prognostic factors are still lacking. The use of purine drugs, such as AZA, in Xanthinuria type II patients requires special attention. Improper use of these drugs can cause serious adverse reactions such as erythematous papules and bone marrow failure. For example, a 38-year-old female patient with cutaneous necrotizing vasculitis who was treated with AZA for 1 month showed a reduction in the whole blood cell count and recovered after replacement with methotrexate (Stiburkova et al., 2018). Another 19-year-old male patient with xanthinuria experienced bone marrow failure after receiving a 3-month course of AZA to treat juvenile idiopathic arthritis. The bone marrow crisis was ameliorated after replacing AZA with trimethoprim/sulfamethoxazole and fluconazole for 3 months (Tanev et al., 2020). *MOCOS* gene polymorphisms are also associated with poor responses to uric acid-lowering therapy with allopurinol drugs (Fanning et al., 2024). These polymorphisms may also influence the metabolism of AZA drugs used in patients with inflammatory bowel disease (Smith et al., 2009). Renal transplant recipients with the *MOCOS* rs594445 single-nucleotide polymorphism can achieve therapeutic effects with lower doses of AZA after surgery (Kurzawski et al., 2012). Purine drugs such as AZA can generate 6-MP through GST *in vivo* through three pathways: (1) phosphorylation to produce the active metabolite 6-thioguanine nucleotide (6-TGN), which enters the nucleus and leads to DNA synthesis obstruction and cell death; (2) methylation by TPMT to form the inactive metabolite 6-methylmercaptopurine (6-MMP); or (3) conversion to 6-thiouracil (6-TU) through XDH or AOX1, which is a catabolic pathway. However, restriction of MOCOS activity directly affects the metabolism and clearance of purine drugs in the body. Moreover, *MOCOS* pathogenic or likely pathogenic variants can reduce the activity of TPMT and decrease the inactive products of 6-MP (Coelho et al., 2016), causing excessive cell death. This is the primary reason for the lack of suitability of purine drugs in patients with *MOCOS* gene deficiencies.

In the MOCOS-KO mouse model, which was constructed using CRISPR/Cas9 technology, the heterozygous mice showed normal long-term growth and development. In contrast, while homozygous mice with deletion showed normal phenotypes at birth, growth retardation appeared at 2 weeks of age, and the mice died at 3–8 weeks of age. The average survival period of these mice was 28.4 days, indicating the possibility of incomplete penetrative premature death. The surviving homozygous deficient mice showed low uric acid levels and elevated concentrations of urinary xanthine and hypoxanthine. The kidneys were the main affected organs, and the pathogenic findings included tubular cystic dilation, deposition of Tamm–Horsfall (uromodulin) protein (THP), renal pelvis lesions, purine stones, upregulation of kidney inflammation, fibrosis, and cell apoptosis. In addition, they showed a reduction in the liver-to-body ratio, abnormal levels of liver function indicators, and an increase in the brain-to-body ratio (Sedda et al., 2021). Goats with homozygous MOCOS p. Asp303Gly variants may show anorexia, weight loss, anemia, renal pelvis dilation, and purine stones in the kidneys and bladder, and die within 2 years of age (Vail et al., 2019). Swiss cattle with homozygous MOCOS p. Ser628ValfsTer9 variant-associated xanthinuria brown present with urolithiasis, purulent bronchopneumonia, renal fibrosis, tubular degeneration, glomerulonephritis, hydronephrosis, and corneal and dermal dysplasia (Jacinto et al., 2023). Mice, cows, and sheep all possess uricase, which can further metabolize uric acid into allantoin, while canine urease is inactivated and uric acid is used as the end-product of purine metabolism. Xanthinuria type II and urinary tract stones (*MOCOS* c.232G>T [splice-site variation], p. Leu46Pro, and p. Ala128GlyfsTer30) have also been reported in dogs (Tate et al., 2021). Reports describing MOCOS variants in mouse models and captive animals presented more severe phenotypes than those reported in clinical cases, which may be attributable to differences in the pathways of purine metabolism end products or undetected clinical cases with more severe phenotypes.

## 5 Conclusion

Patients with Xanthinuria type II usually seek medical attention due to hypouricemia, and genetic sequencing is necessary for diagnosis. Therapeutic management of this condition is primarily based on a low-purine diet. No specific treatment methods are available at present, and the use of purine drugs is contraindicated for patients with Xanthinuria type II since these drugs may cause serious adverse reactions. The p. Cys424Ter variant identified in this study is a new pathogenic variant that has not been reported previously. This disease can be observed in patients of various ages. Some patients may not show notable symptoms in the early stages, although the mouse model showed severe defects. Further research is required to elucidate the clinical profile of Xanthinuria type II and identify the other roles of MOCOS in metabolic pathways.

## Data Availability

The datasets for this article are not publicly available due to concerns regarding participant/patient anonymity. Request to access the datasets should be directed to the corresponding authors.
